# Members of Velvet Complex FpVeA and FpVelB Regulate Asexual Development, Fumonisin Biosynthesis and Virulence in *Fusarium proliferatum*

**DOI:** 10.3390/foods14213666

**Published:** 2025-10-27

**Authors:** Ling Wang, Shaoqing Tang, Weiyang Liao, Zhonghua Sheng, Shikai Hu, Gui’ai Jiao, Gaoneng Shao, Lihong Xie, Peisong Hu

**Affiliations:** State Key Laboratory of Rice Biology and Breeding, China National Rice Research Institute, Hangzhou 311401, China; tangshaoqing@caas.cn (S.T.); 82101215336@caas.cn (W.L.); shengzhonghua@caas.cn (Z.S.); hushikai@caas.cn (S.H.); jiaoguiai@caas.cn (G.J.); shaogaoneng@caas.cn (G.S.); xielihong@caas.cn (L.X.)

**Keywords:** *Fusarium proliferatum*, VeA, VelB, fumonisins, conidiation, virulence

## Abstract

*Fusarium proliferatum* is the causative agent of rice spikelet rot disease, which can produce a group of toxic secondary metabolites, especially fumonisins. Velvet complex is a master regulator governing the development processes and secondary metabolism in filamentous fungi. In this study, we investigated the biological functions of velvet members FpVeA and FpVelB in *F. proliferatum*. Compared with the wild-type Fp9 strain, deletion of *FpveA* or *FpvelB* genes resulted in retarded hyphal growth but promoted conidiation. Disruption mutants exhibited decreased conidial trehalose contents and enhanced sensitivity to H_2_O_2_ stress, as well as inducing expression of photoreceptors. Notably, inactivation of *FpveA* or *FpvelB* led to a reduction in production of fumonisin B1 (FB1), coinciding with downregulation of fumonisin biosynthetic genes. Furthermore, the absence of *FpveA* or *FpvelB* displayed attenuated virulence toward rice spikelets, accompanied by fewer invasive hyphae and a failure of penetration ability. Taken together, these results demonstrated that FpVeA and FpVelB play crucial roles in the asexual development, oxidative stress, toxin synthesis and pathogenicity of *F. proliferatum*.

## 1. Introduction

Fumonisins are a class of polyketide-derived mycotoxin, mainly produced by *Fusarium verticillioides* and *Fusarium proliferatum* [1]. To date, more than 28 analogs have been identified and categorized into A, B, C and P series, of which fumonisin B1 (FB1) is the most abundant and toxic in nature [2]. Long-term and low-dose exposure to FB1 leads to equine leukoencephalomalacia, necrotic enteritis, hepatotoxicity and nephrotoxicity in rats [3]. Consumption of food contaminated with FB1 is epidemiologically associated with high incidences of esophagus cancer in humans and neural tube defects in developing fetuses [4]. The International Agency for Research on Cancer (IARC) classified FB1 as a Group 2B carcinogen to human beings [5]. The European Food Safety Authority (EFSA), the Scientific Committee on Food (SCF), and the Joint FAO/WHO Expert Committee on Food Additives (JECFA) have established the permissible levels of FB1 in food and feed commodities [2,6]. To ensure food security and human health, it is necessary to avoid the entry of fumonisins into the feed and food chain and to minimize their frequency in agricultural commodities.

In filamentous fungi, velvet family proteins share conserved velvet domains, including VeA, VelB, VelC and VosA [7]. In the model organism *Aspergillus nidulans*, VeA physically interacts with VelB to enter into the nucleus in the dark and interact with velvet proteins to form multimeric complexes, such as VelB-VelB, VelB-VosA, VeA-VelB-LaeA and VeA-VelB-VelC [8,9]. Furthermore, VeA and VelB interact with methyltransferase LlmF, heterodimer VapB-VipC, Far1-like DNA binding protein VipA and MpkB kinase in response to environmental stimuli [10,11]. In most cases, VeA and VelB function as global regulators of fungal development and secondary metabolism. In *Aspergillus flavus*, VeA and VelB are involved in the regulation of sporulation, sclerotia formation, stress tolerance and biosynthesis of aflatoxin B1 [12]. In *Aspergillus ochraceus*, VeA and VelB are required for the production of ochratoxin A and pathogenicity in pears [13]. In *Fusarium fujikuroi*, VeA and VelB act as positive regulators for the production of gibberellins, fumonisins and fusarin C, but as negative regulators for bikaverin synthesis [14]. In *Fusarium oxysporum*, VeA and VelB govern conidiation, beauvericin production and virulence in tomatoes [15]. In *Fusarium graminearum*, both VeA and VelB participate in sexual reproduction, biosynthesis of trichothecene and zearalenone and the infection of wheat [16,17]. In *Penicillium chrysogenum*, VelA positively modulates the formation of penicillin and PR toxin, whereas VelB performs an opposite pattern [18]. In *Valsa mali*, disruption of *veA* or *velB* genes results in increases in melanin production, conidiation and sensitivity to abiotic stresses, as well as impaired colonization of apple leaves and twigs [19]. Although the involvement of VeA and VelB in cellular and metabolic processes has been elucidated, it is clear that there are distinct functions in different filamentous fungi.

Rice (*Oryza sativa* L.) is one of the most globally important cereal crops, which provides daily dietary intake for approximately 50% of the world population. Rice spikelet rot disease, predominantly caused by *F. proliferatum*, is one of the most prevalent diseases in rice in China [20]. The epidemiological occurrence of this disease led to enormous yield losses and grain quality deterioration [20,21]. Although chemical fungicides have been implemented to control rice spikelet rot disease, they do not effectively prevent or eliminate the mycotoxin contamination in rice grains. Considering the significance of agricultural welfare and human health, it is imperative to develop management strategies to minimize mold infestations and toxin deposition in the crops. The present study sought to decipher the biological functions of FpVeA and FpVelB (orthologs of VeA and VelB) in *F. proliferatum*. It was found that these two velvet members played versatile roles in vegetative growth, asexual reproduction, host infection and secondary metabolism. These findings provided a novel insight into the development of therapeutic interventions to mitigate mycotoxin risks.

## 2. Materials and Methods

### 2.1. Strains, Media and Culture Conditions

The *F. proliferatum* wild-type Fp9 strain was originally isolated from rice infected with spikelet rot disease [21]. The Fp9 strain was used as the parental strain for genetic transformation. The fungal strains were maintained as conidial suspensions at −80 °C with 25% (*v*/*v*) glycerol. For vegetative growth assays, the strains were routinely cultured on potato dextrose agar (PDA, 200 g/L fresh potato, 20 g/L dextrose and 20 g/L agar) and potato dextrose broth (PDB, 200 g/L fresh potato and 20 g/L dextrose) in darkness. For illumination experiments, cultures were grown in PDB media on a rotary shaker (120 rpm) under white light (7 W/m^2^, 420 lux), provided by four cool-white fluorescent lamps (Philips TLD 18 W/840, Dongguan, Guangzhou, China). For aerial hyphae assays, a sterilized coverslip was inserted into PDA media at an angle of 45 degrees, the aerial hyphae were allowed to be grown on the coverslip, and the hyphal tips were observed under an optical microscope (BX60F-3, Olympus, Tokyo, Japan). For conidiation assays, the strains were inoculated into yeast extract peptone dextrose (YEPD, 10 g/L yeast extract, 20 g/L peptone and 20 g/L dextrose) and mung bean liquid media (40 g/L mung beans) at 28 °C with shaking at 120 rpm. The conidia were harvested by filtration and counted using a hemocytometer. After staining with 10 μg/mL calcofluor white (CFW) solution (Sigma-Aldrich, St. Louis, MO, USA), the conidia were observed using a fluorescent microscope (DM-LB2, Leica, Mannheim, Germany).

### 2.2. Characterization and Phylogenetic Analysis of Proteins

The amino acid sequences of VeA (AAD42946) and VelB (ABQ17967) from *A. nidulans* were used as queries to search for homologs in the database of *F. proliferatum* compiled by the BLASTP algorithm. Putative velvet domain was identified through Conserved Domain Database [22]. Nuclear localization signal (NLS) was predicted by NLStradamus at http://www.moseslab.csb.utoronto.ca/NLStradamus/ (accessed on 16 August 2023). PEST motif was predicted using epestfind at http://emboss.bioinformatics.nl/cgi-bin/emboss/epestfind (accessed on 16 August 2023). The orthologs of VeA or VelB proteins were retrieved from National Center for Biotechnology Information (NCBI) at https://www.ncbi.nlm.nih.gov/ (accessed on 20 September 2023). Multiple sequences alignment was performed using Clustal W program (Larkin et al., 2007) [23]. Phylogenetic tree was constructed using a neighbor-joining method with MEGA12 software (molecular evolutionary genetic analysis, version 12.0, Mega Limited, Auckland, New Zealand) [24]. The bootstrap value was used for 1000 replicates with the p-distance model.

### 2.3. Gene Deletion and Complementation

Deletion and complementation of the targeted genes was conducted with a homologous recombination strategy [25]. To obtain deletion mutants, the upstream and downstream flanking fragments of the genes were amplified from the genomic DNA of the *F. proliferatum* Fp9 strain. The hygromycin phosphotransferase gene (*HYG*) was amplified using plasmid pFGL821 as a template. The fusion cassettes overlapping *HYG* were assembled by double-joint PCR [26]. The resulting constructs were transformed into the protoplasts of the Fp9 strain via polyethylene glycol (PEG)-mediated transformation. The putative transformants were screened with 200 μg/mL hygromycin B (CalBiochem, La Jolla, CA, USA). Diagnostic PCR and Sanger sequencing were used to confirm the homologous integration. Southern blot was further performed using DIG High Prime DNA Labeling and Detection Starter Kit I (Roche Diagnostics, Mannheim, Germany) to detect integration events. To generate the complemented strains, the DNA fragments containing the full-length coding region and its native promoter were amplified and fused with geneticin resistance gene (*GEN*). The complementary constructs were transformed into the protoplasts of the corresponding deletion mutants. Transformants were selected with 200 µg/mL G418 (Solarbio, Beijing, China) and verified by diagnostic PCR. Schematic representations of the construction of gene knockout and complemented strains are illustrated in Figure S1 and Figure S2, respectively. All primers used for genetic manipulation are listed in Table S1.

### 2.4. Trehalose Assay

Trehalose content in conidia was measured according to previously described methods [27]. Three-day-old conidia (2 × 10^8^) were collected from YEPD media, resuspended in sterile water, and disintegrated with glass beads for 5 min at 2000 rpm. The lysate was incubated at 95 °C for 20 min. The supernatant was incubated with an equal volume of 0.2 M sodium citrate (pH 5.5) and 3 mU of trehalase (Sigma-Aldrich, Burlington, MA, USA) at 37 °C for 8 h. Trehalose was hydrolyzed into glucose by trehalase, and the glucose concentration in the supernatant was determined using a Glucose Assay Kit (Sigma-Aldrich, St. Louis, MO, USA). The content of glucose was converted into the amount of trehalose, expressed in μg per 10^7^ conidia.

### 2.5. Measurement of Carotenoid Content

The mycelia were harvested, frozen in liquid nitrogen and ground into powders. Samples were extracted three times with aqueous solution of hexane/acetone (6:4, *v*/*v*) for 30 min each time, until mycelia were colorless. The upper organic phase was collected by centrifugation at 4000 rpm for 10 min. Total carotenoids were measured using Plant Carotenoid Content Assay Kit (Solarbio, Beijing, China) following the manufacturer’s instruction. The amounts of carotenoids were estimated by a UV-Visible spectrophotometer (UV 2550, Shimadzu, Kyoto, Japan) with an absorbance value at 440 nm.

### 2.6. Quantitative Real-Time PCR (qRT-PCR)

Total RNA was extracted with the RNeasy plant mini kit (Qiagen, Hilden, Germany). First-strand cDNA was synthesized by the PrimeScript^TM^ RT reagent Kit with gDNA Eraser (Takara, Kusatsu, Shiga, Japan). The quantitative real-time PCR (qRT-qPCR) was performed using the SYBR Premix Ex Taq^TM^ kit (Takara, Kusatsu, Shiga, Japan) with StepOnePlus Real-Time PCR Systems (Applied Biosystems, Foster City, CA, USA). Relative transcript levels were calculated by the 2^−ΔΔCT^ method [28]. The *β*-tubulin (*Fptub*) gene of *F. proliferatum* was used as an endogenous control for normalization. Expression value in the Fp9 strain was artificially set to one. Three independent technical replicates were performed per sample, and the experiment was carried out with three biological replicates. The primers used for qRT-PCR analysis were shown in Table S2.

### 2.7. Stress Susceptibility Test

The strains were inoculated on PDA supplemented with different concentrations of hydrogen peroxide (H_2_O_2_) or myriocin (Sigma-Aldrich, St. Louis, MO, USA) at 28 °C. The colony diameters were measured after incubation for 4–5 days. Relative growth inhibition was calculated using the formula [(average mycelial diameter of control colonies − average mycelial diameter of stressed colonies)/average mycelial diameter of control colonies] × 100%.

### 2.8. Determination of FB1 Production

FB1 concentration was quantified using high-performance liquid chromatography system (HPLC-1260, Agilent Technologies, Santa Clara, CA, USA) coupled to a tandem mass spectrometry (HPLC-MS/MS) [29]. The strains were grown in PDB media at 28 °C for 9 days in a shaker (120 rpm) in the dark. After centrifugation, the supernatants were homogenized with acetonitrile/water/acetic acid (74:25:1, *v*/*v*/*v*) and filtered through a 0.22 μm nylon membrane. A standard solution of FB1 was purchased from Sigma-Aldrich (St. Louis, MO, USA). Chromatographic separation was achieved on a Zorbax Extend-C18 column (100 × 2.1 mm, 3.5 μm). The injection volume was 2 μL. The mobile phases were made up of 0.1% formic acid in water (phase A) and 0.1% formic acid in methanol (phase B) with a flow rate of 0.2 mL/min. The gradient elution program was performed as follows: 0–1 min, 30% of phase B; 1.01–6 min, linear gradient to 80% of phase B; 6.01–9 min, 80% of phase B; 9.01–10 min, linear gradient to 30% of phase B; 10.01–16 min, 30% of phase B. The temperature of the column oven was kept at 30 °C. The mass spectrometer was operated using selected reaction monitoring (SRM) and electrospray ionization source in positive mode (ESI+). The *m*/*z* transitions of the precursor ion (722.4) and two product ions (352.4 and 334.4) were employed to enhance sensitivity. Capillary voltage was kept at 3500 V. The optimized parameters were used as follows: the source temperature, 120 °C; nitrogen curtain gas pressure, 18 psi; nitrogen nebulizer gas pressure, 40 psi; heating gas pressure, 40 psi. Method performance was determined using the limit of detection (LOD), limit of quantitation (LOQ), linearity, precision, recovery and matrix effect (Table 1). LOD and LOQ were determined based on a signal-to-noise ratio (S/N) of 3 to 10. The recovery was assessed with six parallel measurements of the matrix-matched standards solutions at three concentrations (low, medium and high). The precision was demonstrated as Intra-day repeatability and Inter-day repeatability at medium concentration level. The matrix effect was evaluated by matrix-induced signal suppression and enhancement (SSE). SEE (%) = (slope matrix-matched standard curves/slope solvent standard curves) × 100%.

### 2.9. Pathogenicity and Penetration Assay

Conidia were harvested from YEPD media by centrifugation and adjusted to the desired concentration of 1 × 10^6^ conidia/mL with sterile distilled water. Virulence tests were performed on a susceptible rice variety, Jiahe 218. For rice spikelet assay, 1 mL of conidial suspension was injected into the central section of a spikelet at the booting stage. The inoculated plants were placed in a greenhouse maintained at 25 °C with a photoperiod of 14/10 h(light/dark) and a relative humidity of 80%. Inoculation of each strain was performed three times and ten plants were inoculated in each replicate. The disease symptoms were photographed, and disease indexes were calculated after 21 days of inoculation [30]. The disease index of individual spikelet was evaluated using a 1–9 scale, where 0 = no disease, 1 = lesions limited to the lower 10.0% of the grain, 3 = 10.1–25.0%, 5 = 25.1–50.0%, 7 = 50.1–75.0% and 9 = more than 75.1%. The disease index was expressed as ∑(the number of grains × associated scale)/(total number of grains × largest scale). For the rice floret assay, a 10 μL droplet of conidial suspension was dripped into single flowering floret at the anthesis stage. The infected plants were kept in a controlled chamber at 25 °C under a relative humidity of 80%. Testing of each strain was conducted in triplicate with at least 15 glumes for each replicate. Infected glumes were observed with a scanning electron micrograph (Hitachi Model SU-8010, Hitachi High-Technologies Corporation, Tokyo, Japan) and a transmission electron micrograph (Hitachi Model H-7650, Hitachi High-Technologies Corporation, Tokyo, Japan) as described before [25]. For the penetration behavior test, the strains were grown on PDA media covered with a layer of sterile cellophane membrane. After being cultured at 28 °C for 3 days, the cellophane membrane, together with the colonies, was removed, and the plate was incubated for an additional 3 days to observe whether the colony appeared on the media. Penetration rates (%) were calculated by percentage of colonies showing breakthrough after membrane removal from three independent replicates through measuring 10 plates in each replicate.

### 2.10. Statistical Analysis

Data were expressed as means ± standard deviations from three independent replicates. Statistical analysis was carried out using the one-way analysis of variance (ANOVA) in GraphPad Prism version 10.2.0 (GraphPad Software, San Diego, CA, USA). Student’s *t*-test was used to determine significant differences. Differences were considered statistically significant when *p*-value was less than 0.05 (*p* < 0.05).

## 3. Results

### 3.1. Identification of FpveA and FpvelB in F. proliferatum

**Figure 1:**
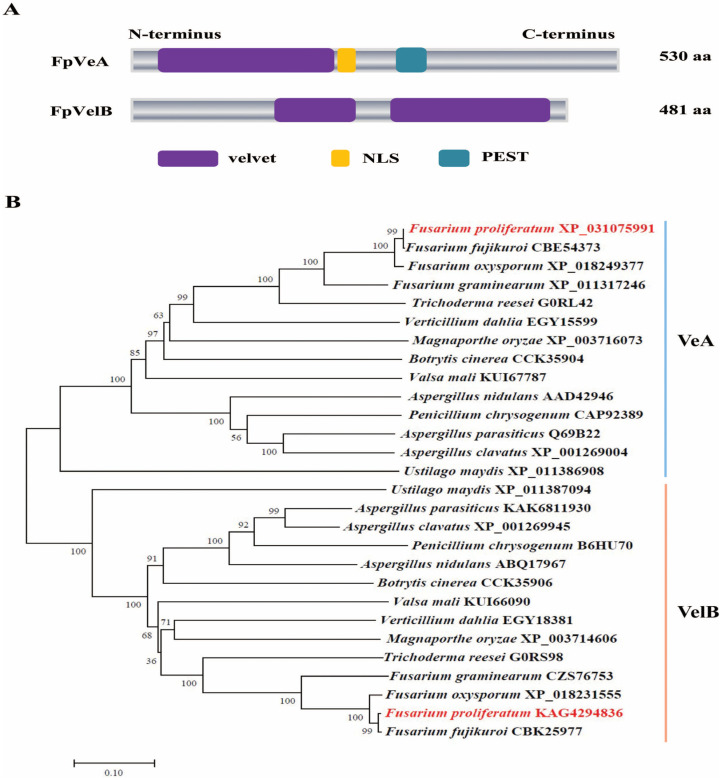
Structure features and phylogenetic analysis of FpVeA and FpVelB proteins. (**A**) Domain architecture of FpVeA and FpVelB in *F. proliferatum*. NLS, nuclear localization signal. PEST, proline (P) glutamic acid (E) serine (S) and threonine (T) rich sequence. (**B**) Phylogenetic relationship of VeA (blue line) and VelB (orange line) from *F. proliferatum* (red) and other different fungal species (black). Phylogenetic tree was constructed by the neighbor-joining method using MEGA12 software. The bootstrap values of 1000 replications are shown at the nodes. The organism names and GenBank accession numbers are indicated on the right of clades. The scale bar represents 0.1 amino acid substitutions per site.

The putative FpVeA and FpVelB in *F. proliferatum* were obtained based on BLASTP (version 2.2.31+) using the orthologs of VeA and VelB in *A. nidulans* as baits. FpVeA contained a velvet domain at the N-terminus, with a nuclear localization signal (NLS) and one putative PEST motif associated with protein degradation (Figure 1A). FpVelB harbored a velvet domain that was interrupted into two segments, lacking an obvious localization sequence and PEST domain (Figure 1A). Phylogenetic analysis showed that VeA and VelB proteins from different filamentous fungi were clustered into two subclades. FpVeA and FpVelB had high levels of sequence similarity with their homologs in other *Fusarium* spp. (Figure 1B). To elucidate the biological function of velvet proteins in *F. proliferatum*, the targeted genes were replaced with a hygromycin-resistance cassette through homologous recombination (Figure S1). The transformants were verified by diagnostic PCR and Southern blot analysis. The complemented strains were generated by reintroduction of the entire gene into the corresponding deletion mutants (Figure S2).

### 3.2. FpVeA and FpVelB Are Involved in Vegetative Growth

**Figure 2:**
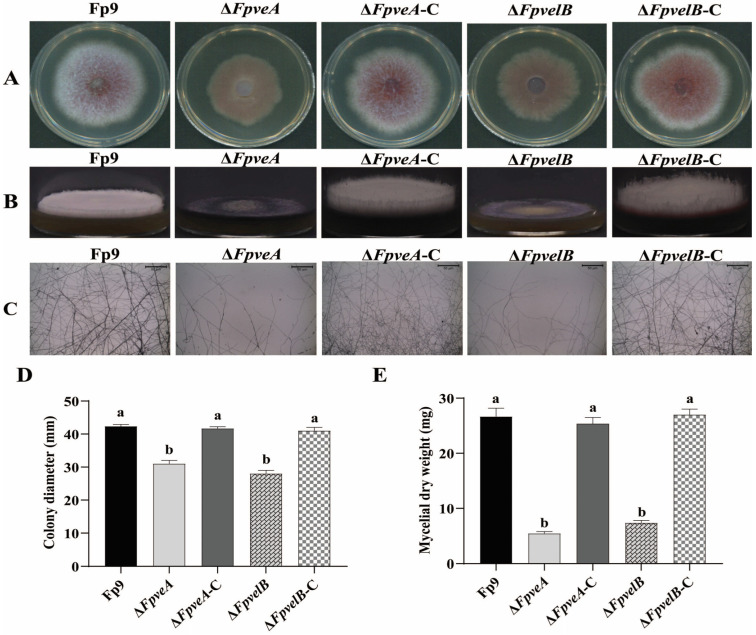
The role of FpVeA and FpVelB on vegetative growth in *F. proliferatum*. (**A**) Colony morphologies of Δ*FpveA* and Δ*FpvelB* on PDA media at 28 °C for 4 days. (**B**) The aerial mycelia of Δ*FpveA* and Δ*FpvelB* in test tubes containing PDA media at 28 °C for 3 days. (**C**) Hyphal tips of Δ*FpveA* and Δ*FpvelB* on the coverslips. Scale = 50 μm (black line). (**D**) Colony diameters of Δ*FpveA* and Δ*FpvelB* on PDA media at 28 °C for 4 days. (**E**) Mycelial dry weights of Δ*FpveA* and Δ*FpvelB* in PDB media at 28 °C for 5 days. Error bars denote standard deviations of three replicates. Different letters above bars represent significant differences determined using ANOVA with Student’s *t*-test (*p* < 0.05). The experiment was repeated three times.

To clarify the impact of FpVeA and FpVelB on fungal growth, all strains were cultured on PDA media. Compared to the Fp9 strain, Δ*FpveA* and Δ*FpvelB* grew more slowly (Figure 2A,D), the aerial mycelia were markedly fewer (Figure 2B) and hyphal tips of the growing colonies were extremely sparse (Figure 2C). After culturing in PDB media, the mycelial biomass of Δ*FpveA* and Δ*FpvelB* was significantly lower than that of the Fp9 strain (Figure 2E). The growth defects were rescued in the complemented strains Δ*FpveA*-C and Δ*FpvelB*-C. These results indicated that FpVeA and FpVelB were essential for vegetative growth in *F. proliferatum*.

### 3.3. FpVeA and FpVelB Negatively Regulate Asexual Sporulation

**Figure 3:**
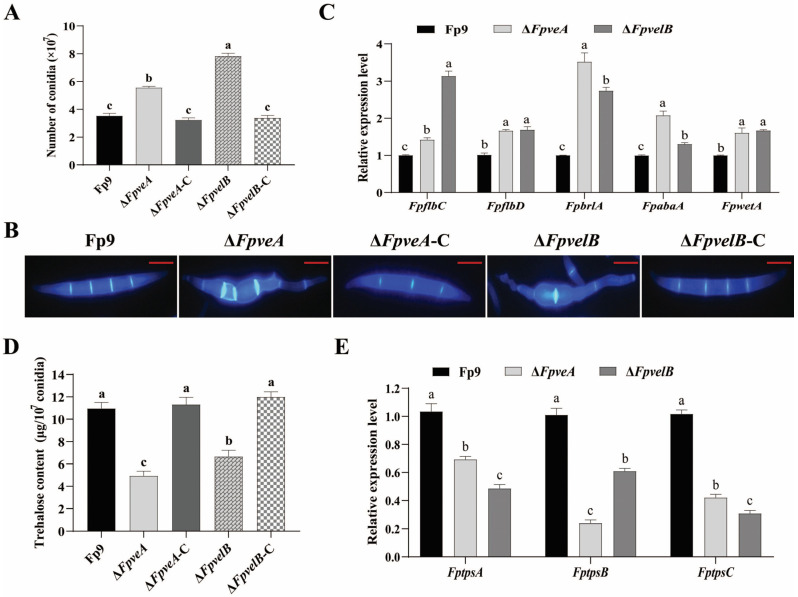
Effect of FpVeA and FpVelB on conidiation in *F. proliferatum*. (**A**) Quantification of conidiospores of Δ*FpveA* and Δ*FpvelB*. After being cultured in YEPD media at 28 °C for 3 days, the conidia were harvested and counted. (**B**) Morphology of macroconidia of Δ*FpveA* and Δ*FpvelB*. Macroconidia were harvested from mung bean media, stained with 10 μg/mL CFW and photographed under a fluorescence microscope. Scale = 5 μm (red line). (**C**) Relative expression levels of spore-specific genes in Δ*FpveA* and Δ*FpvelB*. After being cultured in YEPD media at 28 °C for 3 days, the conidia were harvested for qRT-PCR. *FpflbC* encoded C_2_H_2_ zinc finger transcription factor, *FpflbD* encoded Myb-like transcription factor and *FpbrlA-*, *FpabaA-* and *FpwetA*-encoded regulators were involved in the central development pathway. (**D**) Trehalose contents in the conidia of Δ*FpveA* and Δ*FpvelB*. After being cultured in YEPD media at 28 °C for 3 days, the conidia were collected for the measurement of trehalose contents. (**E**) Relative expression levels of trehalose synthetic genes in Δ*FpveA* and Δ*FpvelB*. After being cultured in YEPD media at 28 °C for 3 days, the conidia were harvested for qRT-PCR. *FptpsA*, *FptpsB* and *FptpsC* encoded α,α-trehalose phosphate synthases. Error bars denote standard deviations of three replicates. Different letters above bars represent significant differences determined using ANOVA with Student’s *t*-test (*p* < 0.05). Each experiment was performed in triplicate.

To ascertain the potential role of FpVeA and FpVelB in conidiation, the strains were cultured in YEPD media. Conidiospores produced by Δ*FpveA* and Δ*FpvelB* were dramatically abundant relative to Fp9 stain, and the number of conidia in Δ*FpvelB* was much higher than that in Δ*FpveA* (Figure 3A). The macroconidia of Δ*FpveA* and Δ*FpvelB* became swollen and abnormal in mung bean media (Figure 3B). The transcript levels of conidiation-specific genes, namely *FpflbC*, *FpflbD*, *FpbrlA*, *FpabaA* and *FpwetA*, were significantly increased in the conidia of Δ*FpveA* and Δ*FpvelB* (Figure 3C). Meanwhile, trehalose contents in the conidia of Δ*FpveA* and Δ*FpvelB* were considerably lower than those in the Fp9 strain, and Δ*FpveA* had lower amounts than Δ*FpvelB* (Figure 3D). The mRNA levels of genes (*FptpsA*, *FptpsB* and *FptpsC*) associated with trehalose synthesis were markedly decreased in Δ*FpveA* and Δ*FpvelB* (Figure 3E). These phenotypes were recovered after genetic complementation with the wild-type genes into the respective deletion mutants. Taken together, these data underscored the repressing effect of FpVeA and FpVelB in conidiophore development in *F. proliferatum*.

### 3.4. FpVeA and FpVelB Are Responsible for Tolerance to Oxidative Stress

**Figure 4:**
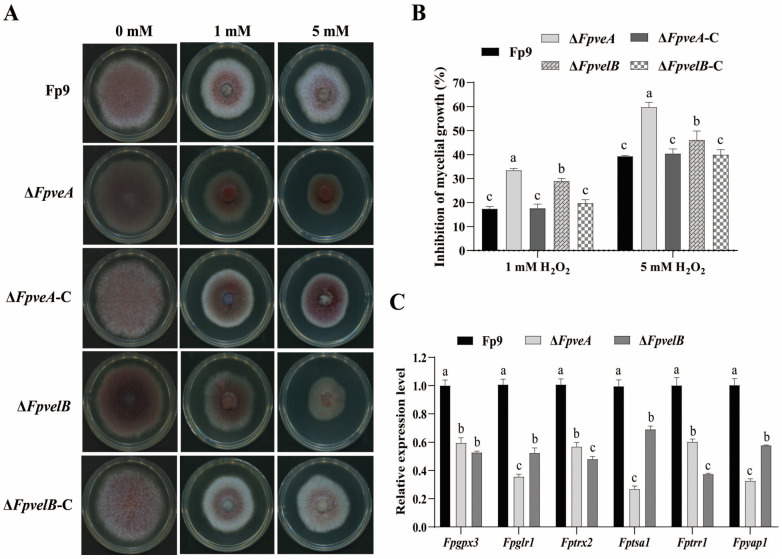
Involvement of FpVeA and FpVelB in the response to oxidative stress. (**A**) Colonies of Δ*FpveA* and Δ*FpvelB* on PDA media supplemented with 1 mM or 5 mM H_2_O_2_ at 28 °C for 5 days. (**B**) Inhibition of mycelial growth of Δ*FpveA* and Δ*FpvelB* on PDA media containing different concentrations of H_2_O_2_. (**C**) Relative expression levels of genes involved in glutaredoxin and thioredoxin systems in Δ*FpveA* and Δ*FpvelB.* After being cultured in PDB media for 3 days, each strain was transferred into PDB supplemented with 5 mM H_2_O_2_ for 45 min and the mycelia were harvested for qRT-PCR. *Fpgpx3* encoded glutathione peroxidase, *Fpglr1* encoded glutathione reductase, *Fptrx2* encoded thioredoxin, *Fptsa1* encoded thioredoxin peroxidase, *Fptrr1* encoded thioredoxin reductase and *Fpyap1* encoded AP1-like transcription factor. Error bars denote standard deviation of three replicates. Different letters above bars represent significant differences determined using ANOVA with Student’s *t*-test (*p* < 0.05). All experiments were repeated three times independently.

As trehalose acted as a protectant against various abiotic stresses, the response of all strains to oxidative stress was evaluated. Δ*FpveA* and Δ*FpvelB* were more sensitive to H_2_O_2_ than the Fp9 strain (Figure 4A). The growth inhibition percentages of Δ*FpveA* and Δ*FpvelB* were significantly higher than those of the Fp9 strain, and the sensitivity of Δ*FpveA* was more obvious than Δ*FpvelB* (Figure 4B). Reintroduction of *FpveA* and *FpvelB* into the corresponding mutants restored the tolerance to oxidative stress. After being exposed to H_2_O_2_ treatment, the mRNA levels of genes related to the glutaredoxin system (*Fpgpx3* and *Fpglr1*), thioredoxin system (*Fptrx2*, *Fptsa1* and *Fptrr1*) and transcription factor *Fpyap1* were dramatically reduced in Δ*FpveA* and Δ*FpvelB* (Figure 4C). Overall, these findings indicated that FpVeA and FpVelB regulated the response of *F. proliferatum* to oxidative stress.

### 3.5. FpVeA and FpVelB Affect Light Perception and Carotenoid Accumulation

**Figure 5:**
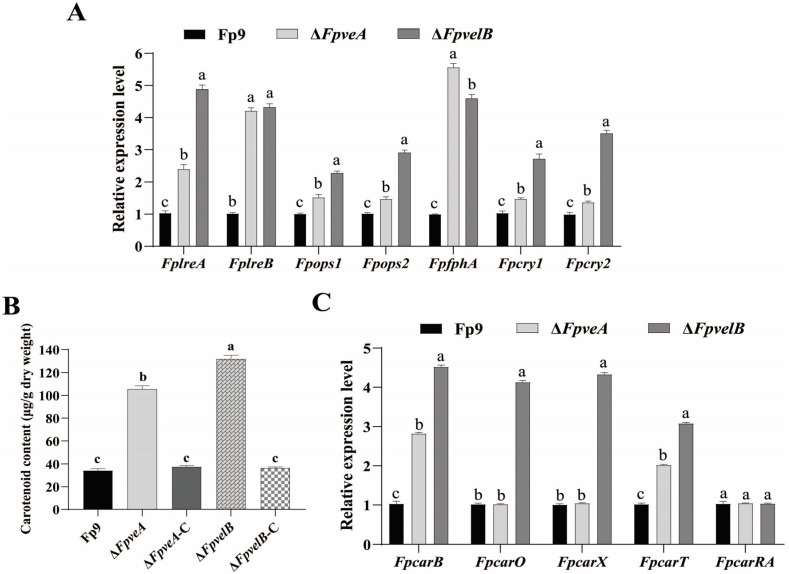
The influence of FpVeA or FpVelB on light perception in *F*. *proliferatum*. (**A**) Relative expression levels of genes encoding photoreceptors in Δ*FpveA* and Δ*FpvelB.* After being cultured in PDB under continuous illumination at 28 °C for 3 days, the mycelia were harvested for qRT-PCR. *FplreA* and *FplreB* encoded white collar proteins, *Fpops1* and *Fpops2* encoded opsins, *FpfphA* encoded phytochrome, and *Fpcry1* and *Fpcry2* encoded cryptochromes. (**B**) Carotenoid contents of Δ*FpveA* and Δ*FpvelB* in PDB media under continuous illumination at 28 °C for 7 days. (**C**) Relative expression levels of genes associated with carotenoid synthesis in Δ*FpveA* and Δ*FpvelB*. After being cultured in PDB under continuous illumination at 28 °C for 3 days, the mycelia were harvested for qRT-PCR. *FpcarB* encoded phytoene desaturase, *FpcarO* encoded opsin-like protein, *FpcarX* encoded β-carotenoid-cleaving oxygenase, *FpcarT* encoded torulene-cleaving oxygenase, and *FpcarRA* encoded phytoene synthase and carotene cyclase. Error bars denote standard deviation of three replicates. Different letters above bars represent significant differences determined using ANOVA with Student’s *t*-test (*p* < 0.05). The experiment was repeated three times.

To assess if FpVeA or FpVelB impacted light absorption, the expression patterns of light-sensing photoreceptors were investigated. Unexpectedly, the mRNA levels of photoreceptors, including blue light receptors (*FplreA* and *FplreB*), green light receptors (*Fpops1* and *Fpops2*), a red light receptor (*FpfphA*) and cryptochromes (*Fpcry1* and *Fpcry2*), were notably increased in ∆*FpveA* and ∆*FpvelB* (Figure 5A). Since carotenoids can be used as indicators of the photoinduction [31], the influence of FpVeA or FpVelB on the carotenogenesis was further analyzed. In comparison, ∆*FpveA* and ∆*FpvelB* had greater contents of the intracellular carotenoids, and ∆*FpvelB* had a more pronounced effect on carotenoid production than ∆*FpveA* (Figure 5B). Except for *FpcarRA*, carotenoid structural genes (*FpcarB*, *FpcarO*, *FpcarX* and *FpcarT*) were upregulated in ∆*FpveA* or ∆*FpvelB* (Figure 5C). The carotenoid concentrations were restored to the levels of the Fp9 strain by recomplementation of *FpveA* or *FpvelB*. Together, these results indicate that FpVeA and FpVelB negatively affected light absorption and carotenogenesis.

### 3.6. FpVeA and FpVelB Contribute to Fumonisin Biosynthesis

**Figure 6:**
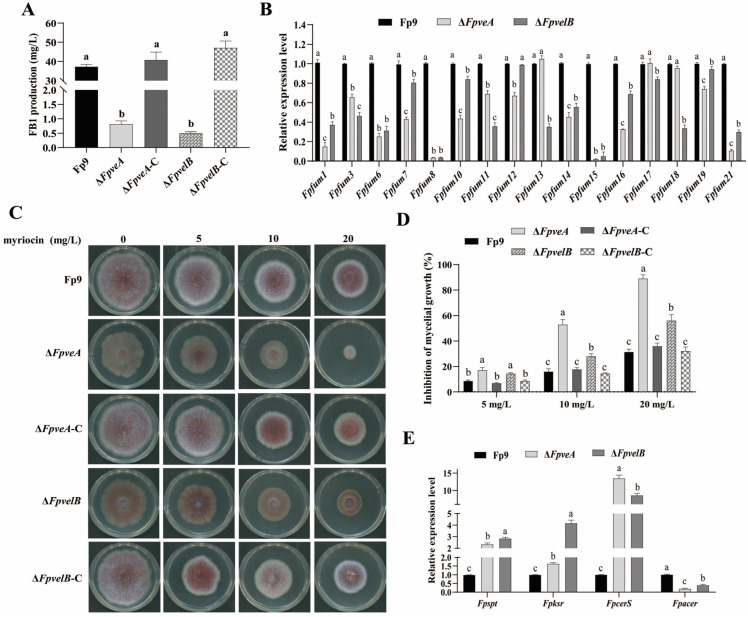
Effect of FpVeA and FpVelB on FB1 production in *F. proliferatum*. (**A**) The amounts of FB1 produced by Δ*FpveA* and Δ*FpvelB* in PDB media at 28 °C for 9 days. (**B**) Relative expression levels of fumonisin biosynthetic genes (*Fpfums*) in Δ*FpveA* and Δ*FpvelB*. After being cultured in PDB at 28 °C for 3 days, the mycelia were harvested for qRT-PCR. (**C**) Colonies of Δ*FpveA* and Δ*FpvelB* on PDA media supplemented with 5, 10 or 20 mg/L myriocin at 28 °C for 4 days. (**D**) Inhibition of mycelial growth of Δ*FpveA* and Δ*FpvelB* under different concentrations of myriocin. (**E**) Relative expression levels of genes responsible for sphingolipid biosynthesis in Δ*FpveA* and Δ*FpvelB*. After being cultured in PDB at 28 °C for 3 days, the mycelia were harvested for qRT-PCR. *Fpspt* encoded serine palmitoyltransferase, *Fpksr* encoded 3-ketosphonganine reductase, *FpcerS* encoded ceramide synthase and *Fpacer* encoded alkaline ceramidase. Error bars denote standard deviations of three replicates. Different letters above bars represent significant differences determined using ANOVA with Student’s *t*-test (*p* < 0.05). Each experiment was carried out with three replicates.

To determine whether FpVeA and FpVelB were involved in fumonisin biosynthesis, FB1 contents of all strains were quantified by HPLC-MS/MS. The levels of FB1 produced by Δ*FpveA* and Δ*FpvelB* were much lower than those produced by the Fp9 strain (Figure 6A). Simultaneously, the mRNA levels of most genes (*Fpfums*) responsible for fumonisin biosynthesis were noticeably decreased in Δ*FpveA* and Δ*FpvelB* (Figure 6B). Specifically, little or no transcripts of *Fpfum8* and *Fpfum15* were detected in both null mutants. The levels of FB1 production were restored by targeted gene complementation. FB1 can competitively inhibit ceramide synthase, and accordingly, we investigated whether or not sphingolipid metabolism could be influenced in null mutants. Lack of *FpveA* or *FpvelB* increased the sensitivity to myriocin, a selective inhibitor of serine palmitoyltransferase (Figure 6C,D). Moreover, the transcripts of *Fpspt*, *Fpksr* and *FpcerS* genes were markedly increased, while expression of the *Fpacer* gene was decreased in Δ*FpveA* and Δ*FpvelB* (Figure 6E). In total, these results revealed that FpVeA and FpVelB were important factors controlling FB1 biosynthesis and sphingolipid metabolism.

### 3.7. FpVeA and FpVelB Are Required for Full Virulence

**Figure 7:**
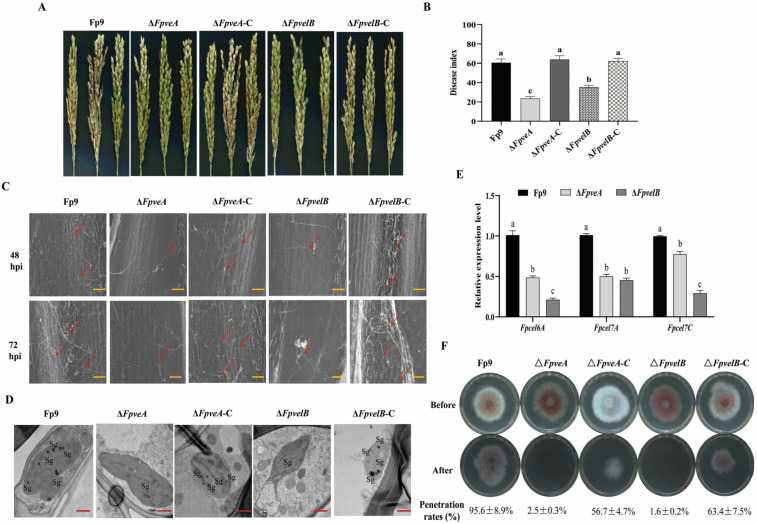
The role of FpVeA and FpVelB on the virulence of *F. proliferatum*. (**A**) Disease symptoms of rice spikelets caused by Δ*FpveA* and Δ*FpvelB* at 21 days post-inoculation (dpi). (**B**) Disease indexes of infected spikelets inoculated with Δ*FpveA* and Δ*FpvelB*. The disease severity of rice spikelets were evaluated at 21 dpi. (**C**) Invasive hyphae of Δ*FpveA* and Δ*FpvelB* on rice glumes at 48 hpi and 72 hpi. Invasive hyphae were observed under scanning electron microscopy. The red arrows indicate invasive hyphae. Scale = 100 μm (orange line). (**D**) Ultrastructure of infected glumes challenged by Δ*FpveA* and Δ*FpvelB* at 72 hpi. Rice glumes were observed under transmission electron microscopy. Sg indicates starch grains in the chloroplasts. Scale = 500 nm (red line). (**E**) Relative expression levels of cellulase-encoding genes of *F. proliferatum* in rice glumes inoculated with Δ*FpveA* and Δ*FpvelB*. *Fpcel6A*, *Fpcel7A* and *Fpcel7C* encoded cellulases. After being incubated for 72 h, the rice glumes were collected for qRT-PCR. (**F**) The penetration ability of Δ*FpveA* and Δ*FpvelB* against cellophane membranes. The strains were grown on PDA media overlaid with a layer of cellophane at 28 °C for 3 days (Before). After removal of the cellophane with the fungal colonies, the plates were incubated for another 3 days (After) to examine the penetration rates (%). Error bars denote standard deviations of three replicates. Different letters above bars represent significant differences determined using ANOVA with Student’s *t*-test (*p* < 0.05). The experiment was performed with three biological replicates.

To characterize the influence of FpVeA and FpVelB on virulence, pathogenicity tests were conducted on rice spikelets at the booting stage. Notably, few lesions appeared on the spikelets caused by Δ*FpveA* and Δ*FpvelB*, and to a lesser extent, Δ*FpveA*, whilst chlorotic and necrotic lesions were developed on the spikelets infected by the Fp9 strain (Figure 7A). Disease severity in the plants inoculated with Δ*FpveA* and Δ*FpvelB* was much lower than in those with the Fp9 strain (Figure 7B). To validate the effect of FpVeA and FpVelB on the onset of infection, the rice florets were inoculated at the anthesis stage. Invasive hyphae of the Fp9 strain extended into the epidermal cells of rice glumes at 48 h post-inoculation (hpi), and the massive filaments established an interconnected hyphal network at 72 hpi (Figure 7C). Conversely, the invasive hyphae of Δ*FpveA* and Δ*FpvelB* were rarely at 48 hpi, and the limited hyphae were attached to the epidermis of the glumes at 72 hpi (Figure 7C). At the same time, the numbers of starch grains in the chloroplasts of rice cells attacked by Δ*FpveA* and Δ*FpvelB* were lower than in those attacked by the Fp9 strain (Figure 7D). In addition, transcript levels of cellulase-encoding genes (*Fpcel6A*, *Fpcel7A* and *Fpcel7C*) were markedly decreased in the rice glumes inoculated with Δ*FpveA* and Δ*FpvelB* (Figure 7E). To further clarify whether the attenuated pathogenicity could be attributed to the penetration behavior, the deletion mutants were cultured on PDA overlaid with cellophane. More clearly, Δ*FpveA* and Δ*FpvelB* failed to penetrate cellophane membranes, while the Fp9 strain was able to invade cellophane and form fungal colonies (Figure 7F). All complementation of deletion mutants with the wild-type genes fully restored the pathogenicity. Collectively, these data imply that FpVeA and FpVelB are necessary for the infection and colonization of host tissues.

## 4. Discussion

As a ubiquitous and notorious ascomycete, *F. proliferatum* not only causes devastating diseases in a wide range of crops but also produces a diverse array of toxic secondary metabolites, which pose a serious risk to human and animal health [21]. Efforts to explore the mechanisms underlying pathogenesis could shed light on the control measures of the mycotoxigenic pathogen. Although the essentiality of the velvet complex has been elucidated in some fungi [32], its roles in *F. proliferatum* remain elusive. To the best of our knowledge, this was the first report of the pleiotropic functions of FpVeA and FpVelB on asexual development, virulence and fumonisin production in *F. proliferatum*.

The asexual spores (conidia) are fundamental to the propagation and dissemination for filamentous fungi [33]. Most strikingly, the conidial yields were enhanced significantly by Δ*FpveA* or Δ*FpvelB*, implying that FpVeA or FpVelB repressed conidiophore development in *F. proliferatum*. Such tendencies were observed from orthologs of VeA or VelB in other organisms, including *A*. *nidulans* [8], *Aspergillus fumigatus* [34,35], *Botrytis cinerea* [36], *F. oxysporum* [15], *Cochliobolus sativus* [37], *V. mali* [19] and *Neurospora crassa* [38]. On the contrary, the formation of conidiophores was positive controlled by VeA or VelB in *A. flavus* [39], *Aspergillus parasiticus* [40], *Magnaporthe oryzae* [41] and *Trichoderma reesei* [42]. Nevertheless, in *F. graminearum*, VeA served as an activator for normal sporogenesis [17,43], whereas VelB negatively impacted conidiation [44]. A possible interpretation of this discrepancy is that VeA and VelB fulfill distinctive functions in asexual reproduction in species-unique manners. Concomitantly, transcript levels of the conidiation-specific genes were increased in Δ*FpveA* or Δ*FpvelB*, including *FpflbC*, *FpflbD*, *FpbrlA*, *FpabaA* and *FpwetA* (orthologs of *flbC*, *flbD*, *brlA*, *abaA* and *wetA* in *A. nidulans*). Among them, the fluffy genes (*flbC* and *flbD*) of upstream signaling cascades manipulate the transition from mycelial growth to asexual conidiation, and the central development pathway (*brlA*→*abaA*→*wetA*) is important for formation and maturation of conidiophores [33]. It appears that hyperactivation of conidiation in deletion mutants of *FpveA* or *FpvelB* might be conferred by the crucial regulators of asexual development. In agreement, deletion of *veA* in *A. nidulans* led to elevated conidiation through upregulated expression of the developmental regulator *brlA* [45]. Additionally, inactivation of *FpveA* or *FpvelB* drastically reduced trehalose contents in conidia, indicating that FpVeA or FpVelB positively affected trehalose accumulation in *F. proliferatum*. These results were consistent with the feature in *A. flavus* where the null mutant of *velB* decreased the level of conidial trehalose [46]. The trehalose is a major non-reducing disaccharide which is required for long-term spore viability [47]. Possibly, perturbation of trehalose synthesis in Δ*FpveA* or Δ*FpvelB* might be accountable for aberrant morphologies of macroconidia, suggesting that breakdown of intracellular trehalose does not support the acquisition of energy during spore formation. It should be noted that FpVelB had a more conspicuous impact on conidiation than FpVeA. A plausible explanation for this phenomenon is that VelB was able to cooperate with other regulatory proteins [48]. In *Aspergillus* spp., VelB interacted with the downstream target VosA to form a heterodimer, which promoted sporulation in conjunction with transcription factor VadA [49]. VelB also coordinated with the upstream regulator FluG of the conidiation cascade to induce conidiation [39]. It can be speculated that FpVeA and FpVelB played overlapping but independent functions on asexual development.

Reactive oxygen species (ROS) are a group of byproducts of aerobic metabolism, such as superoxide anion (O_2_^−^), singlet oxygen (^1^O_2_), hydroxyl radical (·OH) and hydrogen peroxide (H_2_O_2_) [50]. Deletion mutants of *FpveA* or *FpvelB* were more sensitive to H_2_O_2_, thereby underscoring the critical roles of velvet complex in protecting *F. proliferatum* from exogenous oxidative stress. Notably, FpVelB exhibited a stronger tolerance to oxidant than FpVeA, which might be associated with the higher level of trehalose content in Δ*FpvelB*. Indeed, the trehalose was capable of sustaining plasma membrane integrity from oxidative damage [47]. Thus, these results indicated a role of intracellular trehalose in conferring protection against oxidative stress in *F. proliferatum*. Moreover, transcript levels of genes related to the glutaredoxin system (*Fpgpx3* and *Fpglr1*) and thioredoxin system (*Fptrx2*, *Fptsa1* and *Fptrr1*) were decreased in Δ*FpveA* or Δ*FpvelB*. The members of the glutaredoxin and thioredoxin families are the thiol-disulfide oxidoreductases, which play principal roles in maintaining redox homeostasis in eukaryotic subcellular organelles [50]. The Yap1 (ortholog of FpYap1) coordinates the interplay between redox status and glutaredoxin- and thioredoxin-dependent systems [51]. Presumably, deletion of *FpveA* or *FpvelB* increased susceptibility to ROS, at least partially, owing to impaired ROS scavenging modulated by thiol-dependent antioxidant systems. These results are in line with the modulation of VeA or VelB in ROS alleviation in *Cochliobolus heterostrophus* [52], *Curvularia lunata* [53], *V. mali* [19] and *B. cinerea* [36]. However, for *F. proliferatum*, the specific mechanism of how velvet proteins regulate antioxidation needs to be further elucidated.

Photoreceptors utilize chromophores to detect specific wavelengths and convert light energy into the biochemical energy in most fungi [54]. Upon light absorption, the conformation of fungal photoreceptors is altered in the phototransduction pathway [55]. Intriguingly, the transcriptional abundances of light-responsive photoreceptors were significantly increased in Δ*FpveA* or Δ*FpvelB*, including the phytochrome for red light, white collar complex for blue light, cryptochromes for ultraviolet and blue lights and opsins for green light, revealing that FpVeA or FpVelB exert negative controls over the expression of photoreceptors. Previously, the involvement of velvet complex in conidiation and its interdependence with photoreceptors was documented in *A. nidulans*, where phytochrome FphA and white collar protein LreB physically interacted with VeA to induce conidiation [56]. Likewise, the red and blue lights were particularly effective in stimulating asexual reproduction through the participation of FphA and LreA proteins in coordination with the high-osmolarity glycerol (HOG) pathway in *Alternaria alternate* [57]. Thus, it did not rule out the possibility that hyperconidiation in Δ*FpveA* or Δ*FpvelB* was the consequence of the integration of fungal photoreception. Apart from light sensors, the lipophilic terpenoid carotenoids is intimately linked to photosynthesis due to their photoreceptive properties in *F. fujikuroi* [58]. The carotenogenetic reaction has also found to be dependent on photoinduction in other *Fusarium* species, including *Fusarium aquaeductuum, F*. *verticillioides* and *F*. *oxysporum* [59]. In support of these findings, deletion of *FpveA* or *FpvelB* caused enhanced carotenogenesis, triggering transcriptional levels of carotenoid structural genes. In contrast, loss-of-function mutants of *veA* and *velB* impeded the photoinduction of the carotenoid pathway in *N. crassa* [60]. Collectively, it was proposed that FpVeA or FpVelB had inhibitory effects on the output of light sensing and accumulation of carotenoids in *F. proliferatum*.

Fumonisin production was very complex and operated by physiological and environmental cues in hierarchical regimes [2]. *F. proliferatum* is a member of the *Fusarium fujikuroi* species complex (FFSC) [61]. The induction of fumonisins biosynthesis in the FFSC species is not only affected by nutrient situations, such as carbon sources [62], nitrogen starvation [63], sugars and amino acids [64], but also influenced by environmental and abiotic factors, including light [65], external pH [66], oxidative stress [67], temperature and water activity [68]. Moreover, different genotypes of *Fusarium* species or strains possess considerable variability in fumonisin production profiles [69,70,71]. Nevertheless, the elucidation of molecular basis for fumonisin biosynthesisis in *F. proliferatum* is relatively limited. It was worth mentioning that the deletion of *FpveA* or *FpvelB* rendered diminished production of FB1 in *F. proliferatum*. Similarly, the absence of *veA* or *velB* blocked the biosynthesis of fumonisins in *F. fujikuroi* [14] and *F. verticilloides* [72]. The fumonisin biosynthetic genes (*fum*) tended to be located contiguously at the chromosome in *F. proliferatum* [21]. The expression of genes (*Fpfums*) in the cluster responsible for synthesizing fumonisins was markedly decreased in Δ*FpveA* and Δ*FpvelB*. Of particular interest was the case of *Fpfum8* and *Fpfum15* genes, the transcript levels of which were nearly abolished. The *fum8* gene (ortholog of *Fpfum8*) encoding an aminotransferase that was necessary for the condensation of polyketide and alanine to generate 20-carbon aminopolyhydroxyalkyl chain, and the formation of fumonisin backbone was blocked when *fum8* was deleted in *F. verticilloides* [2]. The *fum15* gene (ortholog of *Fpfum15*) encoding a cytochrome P450 monooxygenase that catalyzed hydroxylation of the backbone, and the null mutant of the *fum15* gene impeded production of FB_1_ as a consequence of lacking of the hydroxyl at the C-10 position [73]. It can be inferred that FpVeA and FpVelB participated in the regulation of FB1 biosynthesis. Additionally, FB1 competitively inhibited ceramide synthase (CerS), due to its structural similarity to sphinganine and sphingosine [74]. Sphinganine was produced from L-serine and palmitoyl-CoA catalyzed by serine palmitoyltransferase (SPT) and 3-ketosphonganine reductase (KSR) [2]. Ceramide was synthesized from sphinganine by CerS, then converted into sphingosine by alkaline ceramidase (Acer) [2]. The expression of the *Fpspt*, *Fpksr* and *FpcerS* genes was significantly upregulated, while the *Fpacer* gene was downregulated in Δ*FpveA* or Δ*FpvelB*, which indicated that FpVeA and FpVelB interfered with the metabolism of the sphingolipids, leading to an elevation of free sphingoid bases and depletion of complex sphingolipid bases. Moreover, lack of *FpveA* or *FpvelB* exhibited more sensitivity to myriocin, a specific inhibitor of serine palmitoyltransferase, which is the first and rate-limiting enzyme of de novo sphingolipid biosynthesis [75]. Taken together, these findings highlight that FB1 biosynthesis has a crosstalk with the sphingolipid metabolism orchestrated by velvet proteins in *F. proliferatum*.

The mycotoxins provide advantages for fungal fitness and adaptation to specific environments [76]. As previously mentioned, fumonisins have been regarded as virulence factors, facilitating the infection of *F. proliferatum* in rice [77]. Thus, the reduced production of FB1 caused by Δ*FpveA* and Δ*FpvelB* could partly explain the loss of virulence observed in rice spikelets. The genetic potential to produce mycotoxins mediated by velvet complex might be capable of assisting the pathogens to infect the plants. In rice *bakanae* disease pathogen *F. fujikuroi*, deletion mutants of *veA* or *velB* were unable to cause stem elongation of rice seedlings due to complete blockage of gibberellin production [14]. In wheat pathogen *F. graminearum*, a lack of *veA* or *velB* resulted in a reduction in the levels of trichothecenes, whereby disease severities were alleviated on wheat heads [17,43,44]. In the corn fungus *F. verticillioides*, a knockout mutant of *veA* hindered the synthesis of fumonisins and fusarins, and it was non-pathogenic to maize seedlings [78]. The *veA* null mutant of *C. heterostrophus* failed to generate T-toxins, along with displaying a compromised virulence on maize [52]. The absence of *veA* or *velB* in *Penicillium expansum* led to the inhibition of patulin formation and the loss of the ability to infect apples [79,80]. For *A. ochraceus*, silencing of *veA* and *velB* suppressed ochratoxin production, and the mutants were less aggressive on pear fruits [13]. However, in the gray mold *B. cinerea*, *veA* positively operated host infection, whilst it was not required for production of phytotoxic botcinic acid and botrydial [81]. Although disruption of *veA* blocked synthesis of dothistromin in *Dothistroma septosporum*, it had no apparent effect on disease progression [82]. Further studies are necessary to clarify the mechanisms of velvet proteins in coordinating the toxigenic potential and pathogenicity in plant pathogenic fungi.

*F. proliferatum* initially infects rice florets during anthesis and subsequently infiltrates into the inner tissues of the glumes, ultimately damaging the rice spikelets [83]. Remarkably, inactivation of *FpveA* or *FpvelB* caused severe impairment of the colonization of rice spikelets, accompanied by a restricted expansion of invasive hyphae and failure of penetration ability. These indicate that FpVeA or FpVelB are required for complete invasion into host tissues. Velvet proteins are main drivers in controlling virulence traits in several phytopathogenic fungi. Deletion of *veA* or *velB* leads to decreased pathogenicity of *F. fujikuroi* on rice [14], *F. oxysporum* on tomato [15], *F. graminearum* on wheat [16,43], *Ustilago maydis* on maize [84], *V. mali* on apple [19] and *B. cinerea* on grape [35,85]. To overcome the host’s defense, the hemibiotrophic pathogens possess the ability to depolymerize the plant cell walls by secreting cell-wall-degrading enzymes like hydrolase, pectinase, cellulose, hemicellulase and ligninase [86]. Noteworthy, disruption of *FpveA* or *FpvelB* resulted in downregulation of cellulase-encoding genes in infected glumes. The cellulases belonging to glycoside hydrolases facilitate fungal invasion through degradation of host cellulose [86]. Therefore, FpVeA and FpVelB play roles in aggravating disease development, which might be partially attributable to modulation of cellulose degradation. As a whole, these observations illuminate that FpVeA and FpVelB contributes to the capacity of the pathogen to penetrate and invade plant tissues.

**Figure 8:**
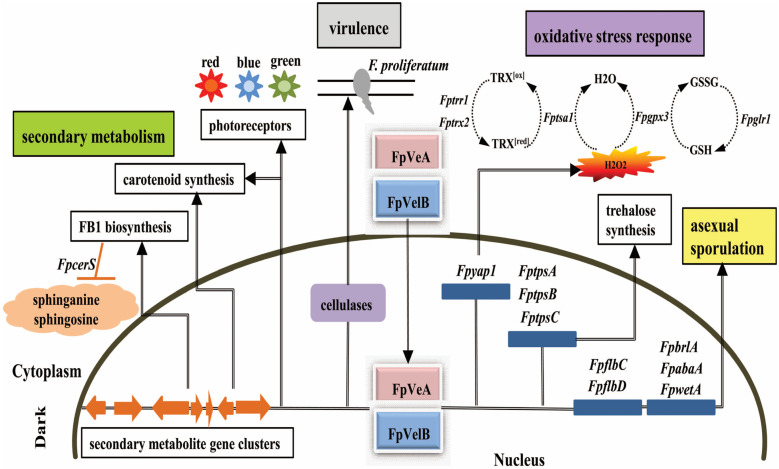
Proposed model for the regulation of FpVeA and FpVelB in asexual development, oxidative stress, FB1 production and virulence in *F. proliferatum*. Gene names are italicized. The following abbreviations are used: TRX^[red]^, reduced thioredoxin; TRX^[ox]^, oxidized thioredoxin; GSH, reduced glutathione; GSSG, oxidized glutathione.

Taken together, we propose a genetic model to illustrate the functional flexibility of FpVeA and FpVelB in *F. proliferatum* (Figure 8). FpVeA enters the nucleus together with FpVelB to form the velvet complex, which consequently regulates multiple aspects of physiology and metabolism in *F. proliferatum*. Firstly, FpVeA and FpVelB suppress conidiation by mediating the development regulatory pathway, but positively control the trehalose synthesis during sporogenesis. Secondly, FpVeA and FpVelB modulate the responses to oxidative stress via the glutaredoxin and thioredoxin systems. Thirdly, FpVeA and FpVelB repress the transcript levels of photoreceptors, concomitantly impeding accumulation of the intracellular carotenoids. Fourth, FpVeA and FpVelB participate in regulation of FB1 production, which is genetically linked to the sphingolipid metabolism. Finally, FpVeA and FpVelB are implicated in plant infection by governing infectious growth, penetration ability and expression of cellulose-encoding genes. Our data corroborates that FpVeA and FpVelB are essential players in asexual development, secondary metabolism and infection processes.

## 5. Conclusions

In summary, this study was the first to reveal the multifaceted roles of FpVeA and FpVelB in *F. proliferatum*. Our findings indicated that two encoded velvet proteins negatively regulated asexual sporulation, but positively governed FB1 production and pathogenicity. It seems likely that velvet complex could be designated as potential targets for antifungal therapeutics, which provides avenues to effectively counteract the detrimental effects of pathogen infection.

## Figures and Tables

**Table 1:** Validation parameters for FB1 quantification with HPLC-MS/MS.

LOD [μg/L]	LOQ [μg/L]	Linear Range [μg/L]	Regression Coefficient (*r*^2^)	Recovery, Low (μg/L)	Recovery, Medium (μg/L)	Recovery, High (μg/L)	Intra-Day Repeatability	Inter-Day Repeatability	SSE
0.513	2.5	2.5–50	0.999	81.8 ± 2.9 (5)	97.5 ± 3.2 (10)	108.4 ± 7.1 (25)	14.0	16.2	116.8

## Data Availability

The original contributions presented in the study are included in the article/Supplementary Material, further inquiries can be directed to the corresponding authors.

## Supplementary Materials

The following supporting information can be downloaded at: https://www.mdpi.com/article/10.3390/foods14213666/s1, Figure S1: Construction and validation of deletion mutants; Figure S2: Construction and validation of complemented strains; Table S1: Primers used for gene deletion and complementation in this study; Table S2: Primers for qRT-PCR in this study.
