# Supplementary material for: Members of Velvet Complex FpVeA and FpVelB Regulate Asexual Development, Fumonisin Biosynthesis and Virulence in Fusarium proliferatum
Source: Foods. 2025 Oct 27;14(21):3666. doi: 10.3390/foods14213666 (PMC12610273; doi:10.3390/foods14213666)
Supplement: Supplementary file 1 [file foods-14-03666-s001.zip › foods-3905388-supplementary.pdf]

Supplementary Materials

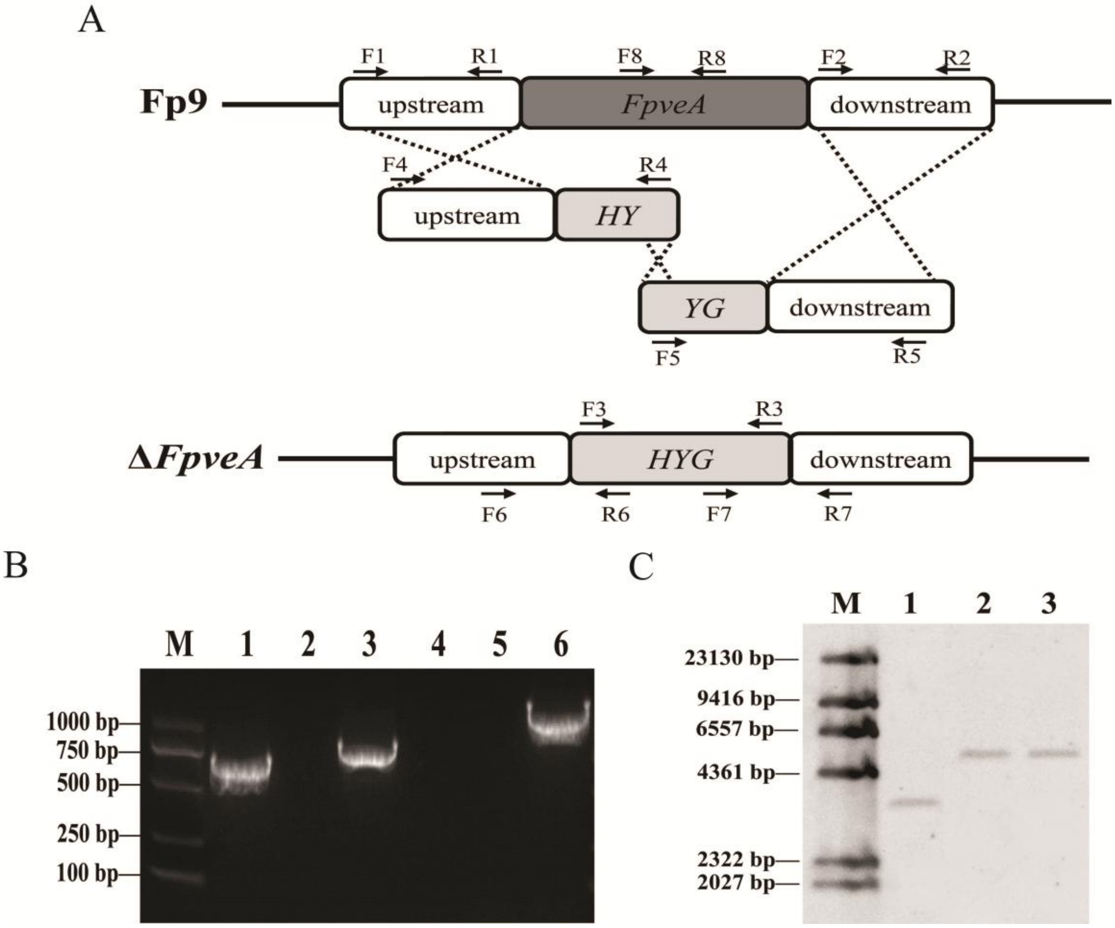

Figure S1. Construction and validation of deletion mutants. (A) Strategy for the disruption of *FpveA* gene. *FpveA* gene was replaced by hygromycin phosphotransferase gene (*HYG*) via homologous recombination. *HY* showed 5′-flanking partial region of *HYG* gene, *YG* showed 3′-flanking partial region of *HYG* gene. Primers positions were indicated with horizontal arrows. (B) Diagnostic PCR for validation of Δ*FpveA*. Homologous integration of the upstream and downstream regions were detected in Δ*FpveA* using primers F6/R6 (lane 1) and F7/R7 (lane 3), whereas none from Fp9 strain (lanes 2 and 4). *FpveA* gene was detected in Fp9 strain with primers F8/R8 (lane 6), but not in Δ*FpveA* (lane 5). M, DL 2,000 DNA marker. (C) Southern hybridization of Δ*FpveA*. The fungal genomic DNA was digested by *BamH*I, electrophoresed, transferred to a nylon membrane, and hybridized with DNA probe. Expected sizes were detected in Fp9 strain (lane 1) and Δ*FpveA* mutants (lanes 2 and 3), respectively. Similar approaches were used to obtain the deletion mutant Δ*FpvelB*.

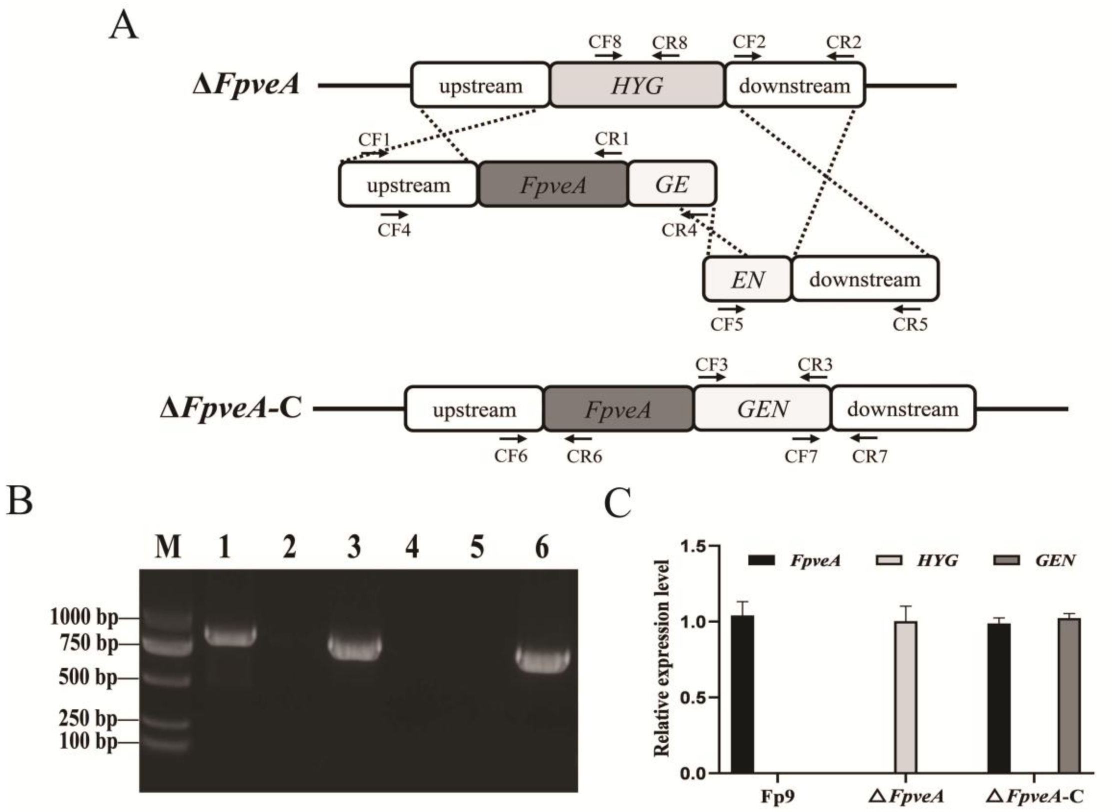

Figure S2. Construction and validation of complemented strains. (A) Strategy for the construction of the complemented strain Δ*FpveA*-C. *GE* showed 5′-flanking partial region of geneticin-resistance gene (*GEN*). *EN* showed 3′-flanking partial region of *GEN* gene. Primers positions were indicated with horizontal arrows. (B) Diagnostic PCR for validation of Δ*FpveA*-C. Homologous integration of the upstream and downstream regions were detected in Δ*FpveA*-C using the primers CF6/CR6 (lane 1) and CF7/CR7 (lane 3), whereas none from Δ*FpveA* (lanes 2 and 4). *HYG* gene was detected in Δ*FpveA* with primers CF8/CR8 (lane 6), but not in Δ*FpveA*-C (lane 5). M, DL 2,000 DNA marker. (C) qRT-PCR analysis of the targeted genes. The relative expression values were normalized against the *β*-tubulin (*Fptub*) gene. Expression of *FpveA* gene in the Fp9 strain was artificially set as 1. Bars in each column denoted standard deviation of three repeated experiments. Similar approaches were used to generate the complementation strain Δ*FpvelB*-C.

Table S1. Primers used for gene deletion and complementation in this study

Primer name	Primer sequence (5′-3′)	Application	Purpose
FpveA-F1	CCTTTTCCTGGCGTTTTCTTCTCGCGACATACGCCCGG	amplify upstream sequence of *FpveA* gene	
FpveA-R1	GCTTTTTCATATACCTACAACTTCAACCTCACTGG	amplify upstream sequence of *FpveA* gene	
FpveA-F2	AAAGAAATAGATTGAATATTAATCACGCGTGTAAG	amplify downstream sequence of *FpveA* gene	
FpveA-R2	ATGCGACTAAAACACGCGACCAGACGAATGTGCTGTTGTC	amplify downstream sequence of *FpveA*gene	
FpveA-F3	TTGTAGGTATATGAAAAAGCCTGAACTCACCGC	amplify *HYG* sequence	Generation of deletion mutant Δ*FpveA*
FpveA-R3	AATATTCAATCTATTTCTTTGCCCTCGGACG	amplify *HYG* sequence	
FpveA-F4	ACCTCTTCCTTGTCCCTC	amplify upstream fragment and *HYG* gene	
FpveA-R4	CGTCCATCACAGTTTGCC	amplify upstream fragment and *HYG* gene	
FpveA-F5	GCATCTCCCGCCGTTCAC	amplify downstream fragment and *HYG* gene	
FpveA-R5	CCCGTCTTGTCTTTTCCC	amplify downstream fragment and *HYG* gene	
FpveA-F6	AATAGCGTCGTAGTGGGTG	Detection of left boarder of deletion mutants	
FpveA-R6	GCAAAGTGCCGATAAACATA	Detection of left boarder of deletion mutants	
FpveA-F7	ATTGACTGGAGCGAGGCG	Detection of right boarder of deletion mutants	
FpveA-R7	GTCCGTGAATGAATTGACAAACTA	Detection of right boarder of deletion mutants	Confirmation of deletion mutant Δ*FpveA*
FpveA-F8	TCTGCGGTCATACAAGTC	Detection of *FpveA* gene of deletion mutants	
FpveA-R8	GAAGAGGGCAAGGACAT	Detection of *FpveA* gene of deletion mutants	
FpveA-F9	ATGACACTGGACCAAATA	Construction of DIG-labeled probe for Southern blot	
FpveA-R9	AACGATCACCCACTACG	Construction of DIG-labeled probe for Southern blot	
FpveA-CF1	TGCCGCGCTCTCATCAGAAGCGACTCGCACTGAGTGCG	amplify upstream sequence and *FpveA* gene	
FpveA-CR1	GTTCTTCTGAATGGCTACACCATCCTCGATTCC	amplify upstream sequence and *FpveA* gene	
FpveA-CF2	GTGTAGCCATTCAGAAGAACTCGTCAAGAAGGC	amplify downstream sequence sequence of *FpveA* gene	Generation of complementary strain Δ*FpveA*-C
FpveA-CR2	AATATTCAATATGGGGATTGAACAAGATGGATTGC	amplify downstream sequence sequence of *FpveA* gene	
FpveA-CF3	CAATCCCCATATTGAATATTAATCACGCGTGTAAG	amplify *GEN* sequence	

FpveA-CR3	GACGCAGTGAGGAAGCGAGACAGACGAATGTGCTGTTGTC	amplify *GEN* sequence	
FpveA-CF4	TCTTTCTCATCTTCCGCA	amplify upstream sequence, *FpveA* and *GEN* gene	
FpveA-CR4	GCAAGGTGAGATGACAGG	amplify upstream sequence, *FpveA*and *GEN* gene	
FpveA-CF5	GCCCCTGATGCTCTTCGT	amplify downstream sequence and *GEN* gene	
FpveA-CR5	GAATAACGAGGACGGTGG	amplify downstream sequence and *GEN* gene	
FpveA-CF6	CGTAGTGGGTGATCGTTT	Detection of left boarder of complementation strains	
FpveA-CR6	GCTCGTCTCTCCTGTTTC	Detection of left boarder of complementation strains	
FpveA-CF7	ATAGCAGCCAGTCCCTTC	Detection of right boarder of complementation strains	Confirmation of complementary strain Δ*FpveA*-C
FpveA-CR7	AGCCAACCAACCATTCAT	Detection of right boarder of complementation strains	
FpveA-CF8	CAGCGTCTCCGACCTGA	Detection of *HYG* gene of complementation strains	
FpveA-CR8	CTCCATACAAGCCAACCAC	Detection of *HYG* gene of complementation strains	
FpvelB-F1	CGCAGCGAGGAAGTGACGCCGCAAGGTAAGGCAGTCGGG	amplify upstream sequence of *FpvelB* gene	
FpvelB-R1	GCTTTTTCATTTGCGTTTTGCGATCAATGATAAGG	amplify upstream sequence of *FpvelB* gene	
FpvelB-F2	AAAGAAATAGAAGAATCTGCGTGAACCATATCACC	amplify downstream sequence of *FpvelB* gene	
FpvelB-R2	CGCGCCGCCTGGCCTCGGTGCTAGTGACTGTCGAGGAGTTGTTG	amplify downstream sequence of *FpvelB* gene	
FpvelB-F3	CAAAACGCAAATGAAAAAGCCTGAACTCACCGC	amplify *HYG* sequence	Generation of deletion mutant Δ*FpvelB*
FpvelB-R3	GCAGATTCTTCTATTTCTTTGCCCTCGGACG	amplify *HYG* sequence	
FpvelB-F4	TGAAGCAACCCCAACACG	amplify upstream fragment and *HYG* gene	
FpvelB-R4	CCATACAAGCCAACCACG	amplify upstream fragment and *HYG* gene	
FpvelB-F5	CATTGGGGAGTTTAGCGA	amplify downstream fragment and *HYG* gene	
FpvelB-R5	GCAGCCGAGCAGTGAGGA	amplify downstream fragment and *HYG* gene	
FpvelB-F6	TACCTACCTCCCCAACCT	Detection of left boarder of deletion mutants	
FpvelB-R6	AGTGCCGATAAACATAAC	Detection of left boarder of deletion mutants	
FpvelB-F7	ATCAGAGCTTGGTTGACG	Detection of right boarder of deletion mutants	Confirmation of deletion mutant Δ*FpvelB*
FpvelB-R7	ATTTGACCTATTTTCCCG	Detection of right boarder of deletion mutants	
FpvelB-F8	GCCCTGCTTATTCTTCC	Detection of *FpvelB* gene of deletion mutants	

FpvelB-R8	CAACCTCTTTACCCGTCT	Detection of *FpvelB* gene of deletion mutants	
FpvelB-F9	AATAAGTCCTGGGATAACG	Construction of DIG-labeled probe for Southern blot	
FpvelB-R9	AGCGAAACAAGACGAGA	Construction of DIG-labeled probe for Southern blot	
FpvelB-CF1	CGCAGCGAGGAAGTGACGCCGCAAGGTAAGGCAGTCGGG	amplify upstream sequence and *FpvelB*gene	
FpvelB-CR1	GTTCTTCTGATCAGTTCTGATCGTACATCTCTTC	amplify upstream sequence and *FpvelB*gene	
FpvelB-CF2	CAATCCCCATAAGAATCTGCGTGAACCATATCACC	amplify downstream sequence sequence of *FpvelB* gene	
FpvelB-CR2	CGCGCCGCCTGGCCTCGGTGCTAGTGACTGTCGAGGAGTTGTTG	amplify downstream sequence sequence of *FpvelB* gene	
FpvelB-CF3	TCAGAACTGATCAGAAGAACTCGTCAAGAAGGC	amplify *GEN* sequence	Generation of complementary strain Δ*FpvelB*-C
FpvelB-CR3	GCAGATTCTTATGGGGATTGAACAAGATGGATTGC	amplify *GEN* sequence	
FpvelB-CF4	TCTTTTTTCCGTCTCTTT	amplify upstream sequence, *FpvelB* and *GEN* gene	
FpvelB-CR4	CTGTCATCTCACCTTGCT	amplify upstream sequence, *FpvelB*and *GEN* gene	
FpvelB-CF5	GTAAAGCACGAGGAAGCG	amplify downstream sequence and *GEN* gene	
FpvelB-CR5	GCCGAGCAGTGAGGAAGT	amplify downstream sequence and *GEN* gene	
FpvelB-CF6	CATCTGGTCTCGTCTTGT	Detection of left boarder of complementation strains	
FpvelB-CR6	AGCGTGTTCGGAATCTTT	Detection of left boarder of complementation strains	
FpvelB-CF7	GGAGCAAGGTGAGATGAC	Detection of right boarder of complementation strains	Confirmation of complementary strain Δ*FpvelB*-C
FpvelB-CR7	CAAAAGCACAAAGTACGG	Detection of right boarder of complementation strains	
FpvelB-CF8	CAGCGTCTCCGACCTGA	Detection of *HYG* gene of complementation strains	
FpvelB-CR8	CTCCATACAAGCCAACCAC	Detection of *HYG* gene of complementation strains	

Table S2. Primers for qRT-PCR in this study

Gene name	Forward sequence (5'-3')	Reverse sequence (5'-3')	Annotation
FpflbC	GGTACTCGCAGCATAGCA	AGGTGTCTCCTGGCTCAT	C2H2 zinc finger transcription factor
FpflbD	CCGTCAAGAACTGGTGGAA	TGGCTGGTGTAGAAATGGATAG	Myb-like transcription factor
FpabaA	TCAGGTGCCAAATGTTCC	GTCAAAGCTGGTCGGGTA	regulator of the central development pathway
FpbrlA	TCGTGAGATGCCTGTCGT	TTCGCAAGTCGGAGAAGA	regulator of the central development pathway
FpwetA	CACCCAGACCTACCTCCCT	CAAGACAAGTGTCGTGCTCC	regulator of the central development pathway
FptpsA	TGACTGCCCTGTATGCT	GCCGTGGTTCTTCTGTT	α,α-trehalose phosphate synthase
FptpsB	TTGAGGACGACACGAAG	TGTGGGTAGTGCTGAACG	α,α-trehalose phosphate synthase
FptpsC	GAACTGGAAGCGAGGTG	GGAAAGCGACGTGTAAGA	α,α-trehalose phosphate synthase
Fpgpx3	CAGTCGGGTCTGTTGGG	ACTCGGGCTTGGTGGTG	glutathione peroxidase
Fpglr1	GCCAGCAGCAAGTTTGG	GCGGCAGCGTTGTAAGT	glutathione reductase
Fptrx2	CGCTTTCAGACAACATCC	TTCCGTTCTTGAAGACAAA	thioredoxin
Fptsa1	ACCGTCAACGACCTCCC	TTCCACCCTCCTGCCATCC	peroxiredoxin
Fptrr1	CCGAGGTGGTGAGGATG	GCCGATGGCGTAGAAGA	thioredoxin reductase
Fpyap1	GACGAGGATGATGAAGATAG	TGGGCTCGGATGTAAGA	AP1-like transcription factor
FplreA	CACGGGTCATATTTCACA	CCAGCTAGAGCATTTCG	white collar-1 protein
FplreB	TTCACCAAGCGAAGAAA	CCAGTGACCTGGAGAATG	white collar-2 protein
Fpops1	ATCGCTCGCCTCTTTGG	GCCGACCTTGACCTTCC	opsin-1 protein
Fpops2	AGAGGTCCAACCACAGG	TGCCAGCAGTAAGAAGAAG	opsin-like protein
FpfphA	CCCTGTAGCAACCAAAC	CATCGCTGTACTCGTCTG	phytochrome
Fpcry1	TTCAAACAATGGTGGGT	GGTCAAACTTCTCGCTCT	cryptochrome
Fpcry2	CCACGGACCTTCTTCTG	GGATTGGGCATGTATTGA	cryptochrome
FpcarB	TGGTGTCGGTGGTGTTT	CCATCGCTGTGGATTAG	phytoene desaturase
FpcarO	AGAGGTCCAACCACAGG	TGCCAGCAGTAAGAAGAAG	opsin-like protein
FpcarX	ACGACCCTGGTAAACCC	CGACACTGATACGCTTGAT	β-carotenoid-cleaving oxygenase

FpcarT	ACGCTACGAAGAAATACTGG	CTGGCGGAGAATGTTGT	torulene-cleaving oxygenase
FpcarRA	GTGAATCTGCCTTGGAT	TGGTGCCAGAAGTGATA	phytoene synthase and carotene cyclase
Fpfum1	CCAACTCTTCTTCCCTGCTA	CACCCTCTACCTCCCACA	polyketide synthase
Fpfum3	CCACGACCGATTTCACAG	TCCAGCCTTCATTTCAGC	dioxygenase
Fpfum6	CTGGAAAGTATGCGGTCAA	GCAGAACTCATCAGCGTCA	cytochrome P450 monooxygenase
Fpfum7	CAGGGCGGTTGACCACTT	GCGACGCCTGGATGTCTT	dehydrogenase
Fpfum8	GCGGAACGAGAAATAGTGA	TGCTGGGTTGAAAGGGAG	aminotransferase
Fpfum10	ACGGAATGACTGAGACGG	GGGAATCGGGTATTGACC	fatty acyl-CoA synthetase
Fpfum11	AAGGTGTTCTCGCCCTCT	ACCCTCCATCACTTTCTCAT	tricarboxylate transporter
Fpfum12	CGACGGCTACACTGCTTA	GCTCCTCACTAGACCCAAA	cytochrome P450 monooxygenase
Fpfum13	TCCGGGCAGCTCAGAATT	GGCGTGATACAGCGACCA	short-chain dehydrogenase/reductase
Fpfum14	TCAAGCTCGCCTCCTACCAC	CCACGATGACCGACTATCCC	peptide synthetase condensation domain
Fpfum15	AACCTCTACCCTATCTTCTGG	TGACTGTCTCCGTACCTGA	cytochrome P450 monooxygenase
Fpfum16	CCTTACAGATGCGTCCCT	ACCGCTTTCCTAATGGTC	fatty acyl-CoA synthetase
Fpfum17	TCTGGAGAAACCTCGAAAGG	ATGCCAATATGCGTGAAATG	longevity assurance factor
Fpfum18	TGATGTGAGGAGCGATGA	TCGAGATTCCTGCCAGCT	longevity assurance factor
Fpfum19	GGAGCCAGATTGGGACAG	ATACCCGAGGAGGAGCAG	ABC transporter
Fpfum21	CGACTGCCAGTATAAAGCC	GTAGCGTAACAGTTTGAGGAG	Zn(II)2Cys6 transcription factor
Fpspt	CGCCAGTGAGACCCTAA	GGCTGTAAACCCAATCA	serine palmitoyltransferase
Fpksr	CCCAAGGGTATTATGAATC	GAAGACATTACATGCCACC	3-ketosphonganine reductase
FpcerS	CGGAAGCACGGAACTAT	CAACAAGGGAACCAACTAG	ceramide synthase
Fpacer	AGGTCAAGGACCCAATT	TACCGACAGGCAAACTC	alkaline ceramidase
Fpcel6A	CCTTGTTATTGAGCCTGAC	GAGTTCCTTAATGGCGTAG	cellulase
Fpcel7A	GGGTCAGAAGCCCAATG	TTGTTGCCAGAAGTAGTGATA	cellulase
Fpcel7C	GGGCTCCGTCGGTATTG	CGCAGGTCTTGCCATCA	cellulase
Fptub	TCGGAAACTCCACCTC	GTCCATACCCTCACCAG	*β*-tubulin
